# Gallic acid improves cardiac dysfunction and fibrosis in pressure overload-induced heart failure

**DOI:** 10.1038/s41598-018-27599-4

**Published:** 2018-06-18

**Authors:** Li Jin, Simei Sun, Yuhee Ryu, Zhe Hao Piao, Bin Liu, Sin Young Choi, Gwi Ran Kim, Hyung-Seok Kim, Hae Jin Kee, Myung Ho Jeong

**Affiliations:** 1 The Second Affiliated Hospital & Yuying Children’s Hospital Wenzhou Medical University, Wenzhou, 325027 China; 2 Heart Research Center of Chonnam National University Hospital, Gwangju, 61469 Republic of Korea; 3 Zhengjiang Rongjun Hospital, 352 Zhongshan road, Jiaxing city, Zhejiang Province 314000 China; 4 Molecular Medicine, BK21 plus, Chonnam National University Graduate School, Gwangju, 61469 Republic of Korea; 5 Hypertension Heart Failure Research Center, Chonnam National University Hospital, Gwangju, 61469 Republic of Korea; 6 The Second Hospital of Jilin University, Changchun, Jilin 130041 China; 7 Department of Forensic Medicine, Chonnam National University Medical School, Gwangju, 61469 Republic of Korea

## Abstract

Gallic acid is a trihydroxybenzoic acid found in tea leaves and some plants. Here, we report the effect of gallic acid on cardiac dysfunction and fibrosis in a mouse model of pressure overload-induced heart failure and in primary rat cardiac fibroblasts, and compare the effects of gallic acid with those of drugs used in clinics. Gallic acid reduces cardiac hypertrophy, dysfunction, and fibrosis induced by transverse aortic constriction (TAC) stimuli *in vivo* and transforming growth factor β1 (TGF-β1) *in vitro*. It decreases left ventricular end-diastolic and end-systolic diameter, and recovers the reduced fractional shortening in TAC. In addition, it suppresses the expression of atrial natriuretic peptide, brain natriuretic peptide, skeletal α-actin, and β-myosin heavy chain. Administration of gallic acid decreases perivascular fibrosis, as determined by Trichrome II Blue staining, and reduces the expression of collagen type I and connective tissue growth factor. However, administration of losartan, carvedilol, and furosemide does not reduce cardiac dysfunction and fibrosis in TAC. Moreover, treatment with gallic acid inhibits fibrosis-related genes and deposition of collagen type I in TGF-β1-treated cardiac fibroblasts. These results suggest that gallic acid is a therapeutic agent for cardiac dysfunction and fibrosis in chronic heart failure.

## Introduction

Heart failure is a major health concern worldwide, and it is a leading cause of morbidity and mortality in the United States and in other developed countries. For example, an estimated 6.5 million Americans over 20 years of age experienced heart failure, on the basis of data from NHANES 2011 to 2014^1^. Heart failure is a common terminal clinical feature in various heart diseases, including hypertension, myocardial infarction, and valvular disease. There are several animal heart failure models, including that of transverse aortic constriction (TAC), left anterior descending ligation, and the aged spontaneously hypertensive rat. TAC mimics human aortic stenosis, and it induces cardiac hypertrophy and heart failure in animals^2^. In addition, TAC induces inflammatory and fibrotic response^3^.

Heart failure is characterized by interstitial fibrosis, chamber remodeling, and reduced ventricular compliance. Activated cardiac fibroblasts lead to excessive deposition of extracellular matrix (ECM) proteins such as collagen type I and fibronectin, and cardiac fibroblasts are major contributors to the progression of heart failure^4^. Cardiac fibroblasts are converted to myofibroblasts, an activated form, in response to pathological stimuli^5^. Collagen type I is a representative ECM expressed in cardiac fibroblasts, the epicardium, adventitia of large vessels, and valve interstitial cells. Transforming growth factor β1 (TGF-β1) activates fibroblasts^6^ and enhances the synthesis of ECM proteins^7^. Connective tissue growth factor (CTGF), a cysteine-rich growth factor, is increased by TGF-β1 and implicated in ECM protein synthesis^8^.

Diuretics, angiotensin-converting enzyme inhibitors, angiotensin receptor blockers (ARBs), and beta-blockers are clinically used to treat heart failure. Recently, selective sinus node inhibitors ivabradine and LCZ696 (valsartan/sacubitril) are attracting attention as therapy for heart failure. Despite effective therapies targeted at improving or treating heart failure, the 5-year mortality rate of this condition ranges from 45% to 60%^9^. This has led many researchers to develop new and safe drugs to improve outcomes in patients with heart failure.

Chinese herbal medicine has also been used for the treatment of chronic heart failure^10^. In animal experiments, several phytochemicals such as curcumin and resveratrol (from grapes) have been demonstrated as a novel potential therapy for heart failure^11,12^. Gallic acid is a 3,4,5-trihydroxybenzoic acid found in tea leaves and some fruits. Recently, there has been increasing evidence that gallic acid confers several benefits on vascular calcification^13^, cardiac hypertrophy and fibrosis^14^, hypertension^15^, and oxidative stress^16^. Heart failure is associated with cardiac hypertrophy and hypertension; hence, we hypothesized that gallic acid can improve heart failure. Here, we investigated the effect of gallic acid in a mouse model of TAC-induced heart failure and in cultured rat fibroblast cells. We compared the effect of gallic acid with those of the ARB losartan, beta-blocker carvedilol, and diuretic furosemide. Our findings demonstrate that gallic acid can be a novel therapeutic agent to treat cardiac dysfunction and fibrosis in heart failure.

## Materials and Methods

### TAC and experimental groups

All animal procedures were approved by the Animal Experimental Committee of Chonnam National University Medical School (CNU IACUC-H-2017-84), and carried out according to the Guide for the Care and Use of Laboratory Animals (US National Institutes of Health Publications, 8^th^ edition, 2011). CD-1 male mice (6 week old, weighing 30 g) were anesthetized using an intraperitoneal injection of ketamine (120 mg/kg) and xylazine (6.2 mg/kg). Mice underwent either sham operation or TAC. TAC was performed as follows. The endotracheal tube of the mice was connected to a rodent ventilator. After exposure of the aortic arch, the thymus was removed. The transverse aortic arch was ligated (using a 7-0 silk suture) between the brachiocephalic and left common carotid arteries with an overlaying 27 G needle. The sham mice underwent the same operation, except for ligation of the aorta. The TAC procedure was confirmed by echocardiography. After 8 weeks, TAC resulted in heart failure. The experimental rats were divided into six groups: sham group (n = 12), TAC group (n = 16), TAC + gallic acid (n = 13, 100 mg/kg/day), TAC + losartan (n = 10, 3 mg/kg/day), TAC + carvedilol (n = 11, 1 mg/kg/day), and TAC + furosemide (n = 6, 3 mg/kg/day). Gallic acid, losartan, carvedilol, and furosemide were dissolved in DMSO. The dosage of gallic acid (100 mg/kg/day) used for *in vivo* experiments was chosen because of the lack of toxicity and tolerable concentration in previous studies^14,15,17^^,^^18^. After drug administration for 2 weeks, the mice were sacrificed.

### Echocardiography

Echocardiography was performed using a Vivid S5 echocardiography system (GE Healthcare, Chicago, IL, USA) with a 13-MHz linear array transducer. Mice were anesthetized using tribromoethanol (Avertin, 114 mg/kg intraperitoneal injection) before the procedures. M-mode (2-D guided) images and recordings were acquired from the long-axis view of the left ventricle at the level of the papillary muscles. The thickness of the anterior and posterior wall was measured from the images, whereas the left ventricular end-diastolic diameter (LVEDD) and left ventricular end-systolic diameter (LVESD) were measured from the M-mode recordings. Fractional shortening (FS) was calculated as FS (%) = (LVEDD − LVESD) × 100/LVEDD.

### Trichrome II Blue staining and morphometric measurement

The paraffin-embedded tissues were cut into 3-µm thick sections, deparaffinized with xylene, and then rehydrated with different grades of alcohol. Trichrome II Blue staining kit (860-013; Ventana Medical Systems, Inc. Strasbourg, France) was used on the BenchMark Special Stains automated slide staining instruments. Trichrome II Blue staining kit is a modification of Masson’s Trichrome Stain. Briefly, heart sections were incubated with Bouin’s solution to intensify the final coloration. Cytoplasm and muscle were stained with Trichrome Red, containing Biebrich scarlet and acid fuchsin, while nuclei were stained with iron hematoxylin. After application of Trichrome Mordant, the collagen was stained with Trichrome Blue II, which contains aniline blue. Trichrome Clarifier, an acetic acid solution, was applied to create a more delicate and transparent shade of color in the heart tissue section. To determine the development of vessel in the hearts of mice in the TAC group, the circumference of the vessels was measured using the NIS Elements Software (Nikon Eclipse 80*i*; Nikon Corp., Tokyo, Japan).

### Reagents

Gallic acid (G7384) was purchased from Sigma (Billerica, MA, USA). Carvedilol (S1831), losartan potassium (S1359), and furosemide (S1603) were purchased from Selleck Chemicals (Houston, TX, USA).

### Primary neonatal cardiac fibroblast cell culture

Primary rat neonatal cardiac fibroblasts were isolated from 1-day-old Sprague–Dawley rat pups (30 pups/preparation). Atrium was removed from the hearts and minced to pass through a 10 mL pipette tip. The heart tissue was digested with collagenase II and pancreatin in 1× ADS buffer (116 mM NaCl, 20 mM HEPES, 10 mM NaH_2_PO_4_, 5.5 mM glucose, 5 mM KCl, 0.8 mM MgSO_4_) at 37 °C on a shaker at 120 rpm for 2 h. After digestion, the cells were centrifuged at 1,000 rpm for 5 min. Fibroblasts were maintained in Dulbecco’s modified Eagle’s medium (DMEM) supplemented with 10% fetal bovine serum (FBS) plus 1× antibiotic-antimycotic mixture. Cells were incubated at 37 °C in 5% CO_2_. Fibroblasts were used from passage one (P1) to two (P2).

### Real-time reverse transcription-polymerase chain reaction (RT-PCR)

Total RNA from heart tissue was isolated with the TRIzol reagent (Invitrogen Life Technologies, Carlsbad, CA, USA), and 1 μg of RNA was used for the reverse transcription reaction with TOPscript RT DryMIX (Enzynomics, Daejeon, South Korea). mRNA levels were quantified with the SYBR Green PCR kit (Enzynomics). The PCR primers used in this study are shown in Table 1.

**Table 1:** Primers for reverse transcription-polymerase chain reaction (RT-PCR).

Gene	Primer sequence (5′ to 3′)
*GAPDH (mouse)*	F: GCATGGCCTTCCGTGTTCCT R: CCCTGTTGCTGTAGCCGTATTCAT
*Collagen type I (mouse)*	F: GAGCGGAGAGTACTGGATCG R: GCTTCTTTTCCTTGGGGTTC
*SMA (mouse)*	F: ACTGGGACGACATGGAAAAG R: AGAGGCATAGAGGGACAGCA
*Fibronectin (mouse)*	F: GATGCACCGATTGTCAACAG R: TGATCAGCATGGACCACTTC
*CTGF (mouse)*	F: CAAAGCAGCTGCAAATACCA R: GGCCAAATGTGTCTTCCAGT
*P8 (mouse)*	F: CCTTCCCAGCAACCTCTAAA R: GAACTTGGTCAGCAGCTTCC
*MMP2 (mouse)*	F: ATGCCATCCCTGATAACCTG R: TGTGCAGCGATGAAGATGAT
*MMP9 (mouse)*	F: GAAGGCAAACCCTGTGTGTT R: AGGAAGACGAAGGGGAAGAC
*MMP13 (mouse)*	F: CCAGAACTTCCCAACCATGT R: GTCTTCCCCGTGTTCTCAAA
*β-MHC (mouse)*	F: GCATTCTCCTGCTGTTTCCT R: CCCAAATGCAGCCATCTC
*α-MHC (mouse)*	F: ATAAAGGGGCTGGAGCACTG R: GCCTCTAGGCGTTCCTTCTC
*GAPDH (rat)*	F: AACCCATCACCATCTTCCAGGAGC R: ATGGACTGTGGTCATGAGCCCTTC
*Collagen type I (rat)*	F: ACCCCAAGGAGAAGAAGCAT R: AGGTTGCCAGTCTGTTGGTC
*SMA (rat)*	F: GACACCAGGGAGTGATGGTT R: GTTAGCAAGGTCGGATGCTC
*Fibronectin (rat)*	F: GAGCGGAGAGTACTGGATCG R: GCTTCTTTTCCTTGGGGTTC
*CTGF (rat)*	F: CCTTGGTGCTCCTCCTCTG R: AGTCGCAGAAGAGACCCTTG

### Western blotting

Total protein from heart tissues was extracted using a lysis buffer (RIPA, 150 mM NaCl, 1% Triton X-100, 1% sodium deoxycholate, 50 mM Tris-HCl pH 7.5, 2 mM EDTA, 1 mM PMSF, 1 mM DTT, 1 mM Na_3_VO_4_, and 5 mM NaF) containing a protease inhibitor cocktail (Calbiochem, EMD Millipore, Billerica, MA, USA). Proteins were subjected to SDS-PAGE and transferred to polyvinylidene difluoride (PVDF) membranes. The membranes were blocked with 5% skim milk in TBST buffer (20 mM Tris, 200 mM NaCl, and 0.04% Tween 20) for 1 h at 25 °C. The membranes were incubated overnight at 4 °C with primary antibodies against collagen type I, fibronectin, CTGF, SMA, ANP, BNP, and GAPDH. They were then incubated with the anti-rabbit or anti-mouse horseradish-peroxidase-conjugated secondary antibodies (1:5000) for 1 h at room temperature. Protein bands were visualized using Immobilon Western detection reagents (EMD Millipore). The Bio-ID software was used to quantify protein expression (Vilber Lourmat, Eberhardzell, Germany).

### Fluorescence immunocytochemistry

Immunocytochemistry was performed as described previously^15^. Rat neonatal cardiac fibroblasts were seeded and serum starved overnight. The cells were pretreated with TGF-β1 (5 ng/mL) for 3 h and then with gallic acid (100 μM) for further 9 h. The cells were fixed with 4% paraformaldehyde, permeabilized with 0.2% Triton X-100, blocked with 3% normal goat serum, and incubated overnight with anti-collagen type I antibody (1:200, Abcam, Cambridge, UK) at 4 °C. They were then probed with goat anti-rabbit IgG secondary antibody, Alexa Fluor 568 conjugate (1:400, Invitrogen). DAPI (4′,6-diamidino-2-phenylindole) was used for nuclear staining. The cells were then observed under a fluorescence microscope (Nikon Eclipse 80*i*; Nikon Corp., Tokyo, Japan). To compare the fluorescence intensity of collagen stain between the groups, the normalized mean intensity was calculated by an automated “measure tool” using the NIS Elements AR 3.0 program (Nikon, Japan).

### Statistical analysis

All data are expressed as means ± standard errors (SE). Differences between data were analyzed by one-way analysis of variance (ANOVA) with the Bonferroni *post hoc* test using GraphPad Prism version 5, and a value of *P* < 0.05 was considered statistically significant.

## Results

### Gallic acid improves cardiac dysfunction in TAC-induced heart failure

To investigate whether gallic acid can ameliorate heart failure, we evaluated cardiac function by echocardiography in mice in the TAC group. We evaluated heart function every 2 weeks and confirmed reduction of FS and increased LV lumen diameter at 8 weeks after the TAC operation (Supplementary Fig. 1A–C). Next, we randomized mice (n = 64) to 5 treatment groups including vehicle, gallic acid, losartan, carvedilol, and furosemide, except for those exceeding average LVEDD and FS values.

**Figure 1:**
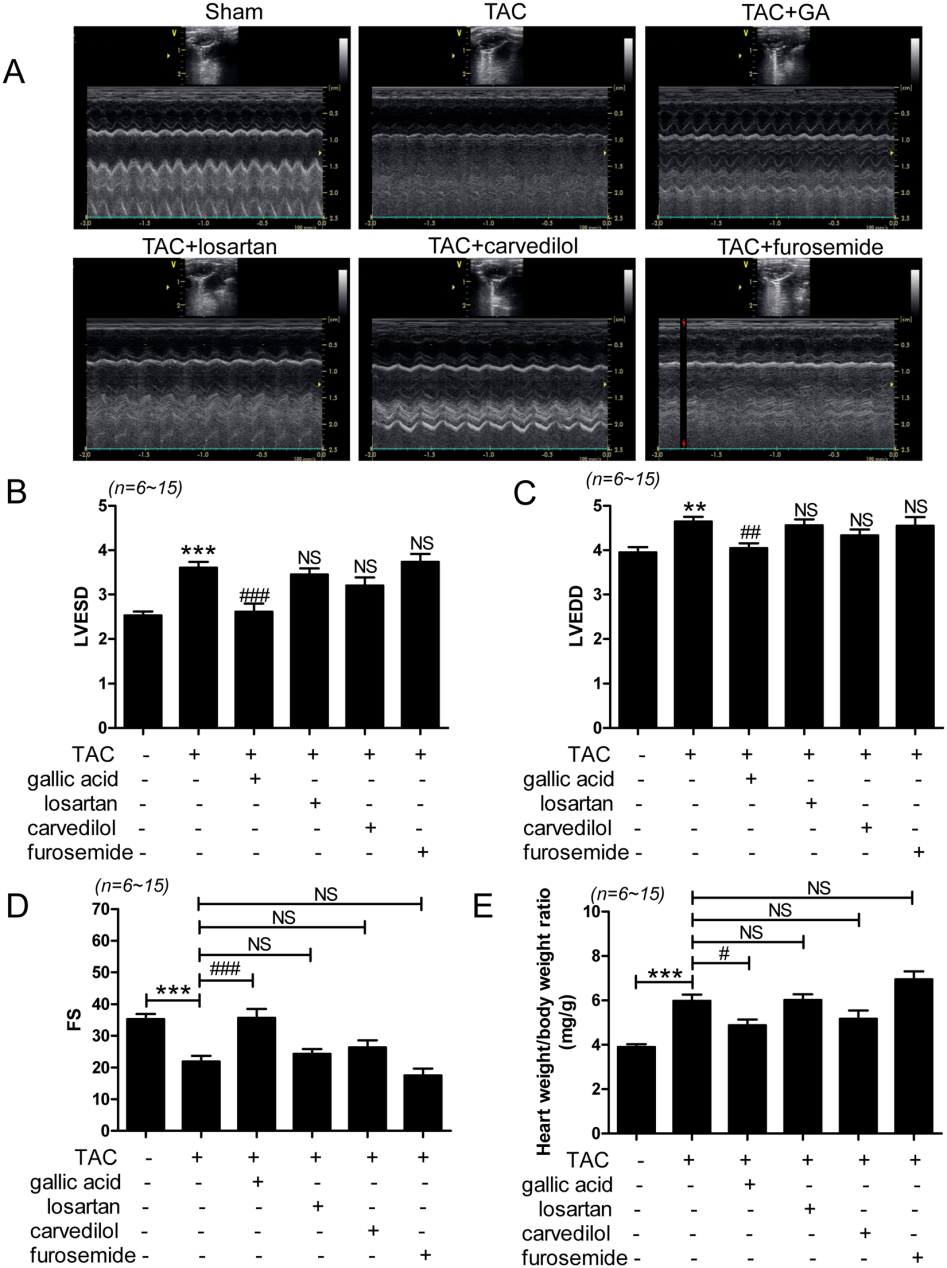
Gallic acid improves cardiac dysfunction in mice with transverse aortic constriction (TAC)-induced heart failure. Heart failure was induced 8 weeks after TAC surgery in mice, after which they were administered either gallic acid (100 mg/kg/day), losartan (3 mg/kg/day), carvedilol (1 mg/kg/day), or furosemide (3 mg/kg/day) for 2 weeks. (**A**) Representative B-mode and M-mode echocardiograms 10 weeks after TAC surgery: sham (n = 12), TAC (n = 16), TAC + gallic acid (n = 13), TAC + losartan (n = 10), TAC + carvedilol (n = 11), and TAC + furosemide (n = 6); (**B**) left ventricular end-systolic diameter (LVESD); (**C**) left ventricular end-diastolic diameter (LVEDD); and (**D**) fractional shortening (FS, %); (**E**) Heart weight to body weight (mg/g) ratio at 10 weeks after sham or TAC treatment with drugs. **P < 0.01 and ***P < 0.001 versus the sham group; ^#^*P* < 0.05, ^##^*P* < 0.01, and ^###^*P* < 0.001 versus the TAC group; NS: not significant.

Echocardiography indicated that TAC considerably increased the LVESD and LVEDD. Treatment with gallic acid, but not losartan, carvedilol, or furosemide, significantly reduced LVESD and LVEDD in TAC (Fig. 1A–C). In addition, gallic acid treatment restored the reduced FS to the level observed in mice in the sham group (Fig. 1D). However, the other drugs failed to recover FS (Fig. 1D). Heart rate was reduced in TAC mice compared to sham mice and was increased by gallic acid treatment (Supplementary Fig. 2). Gallic acid also affected the increased heart mass in TAC. As shown in Fig. 1E, the heart weight/body weight ratio was significantly increased in TAC mice compared with the sham group. Gallic acid treatment decreased the ratio but other drugs did not.

### Gallic acid reduces the expression of heart failure marker genes in TAC-induced heart failure

**Figure 2:**
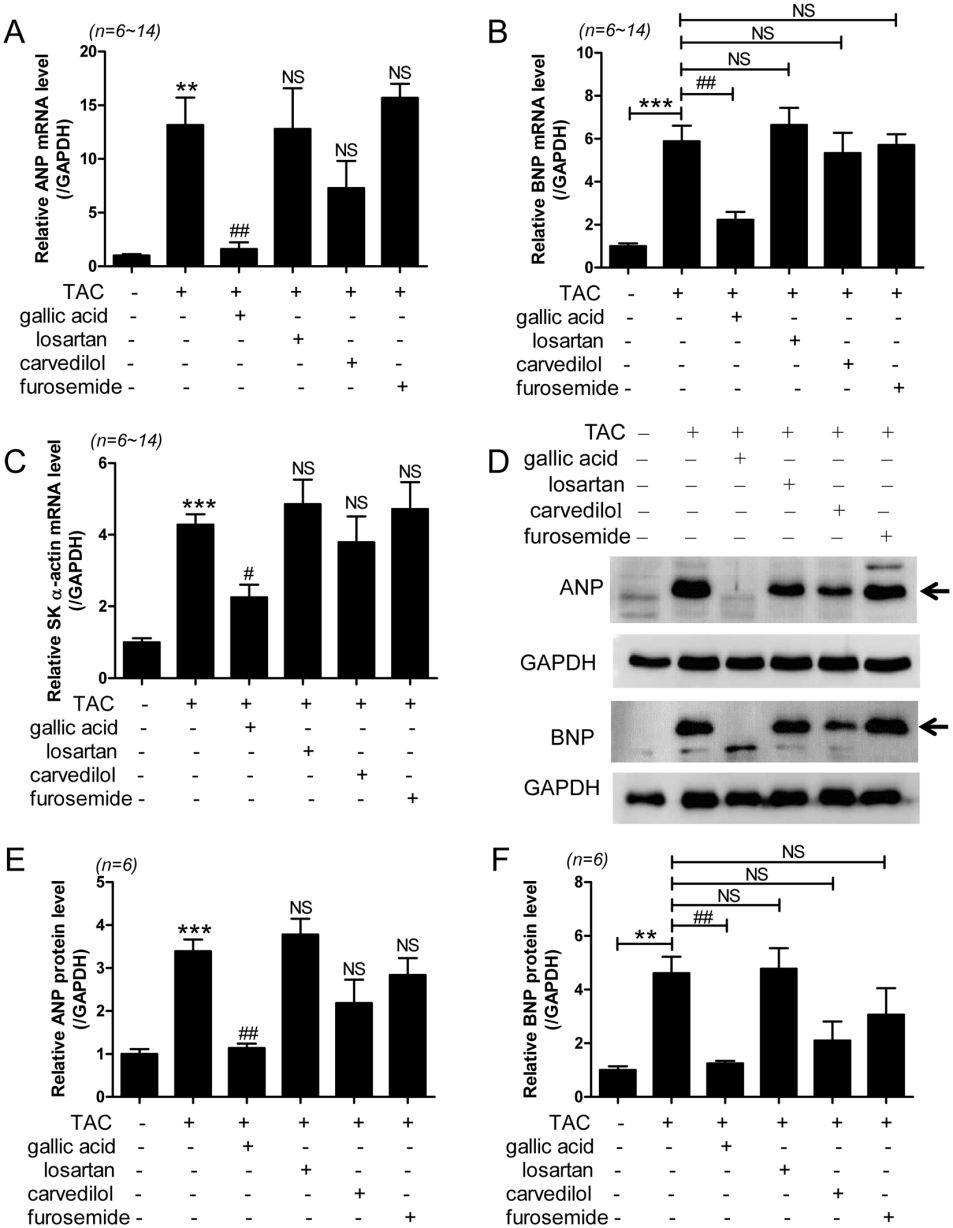
Gallic acid reduces expression of heart failure marker genes in TAC-induced heart failure. Heart failure marker genes were analyzed by RT-PCR in the heart tissues of mice in the TAC group treated with vehicle, gallic acid, losartan, carvedilol, or furosemide. Transcripts for ANP (**A**), BNP (**B**), and skeletal α-actin (**C**) were determined. RNA levels were normalized to GAPDH. Data are means ± SE. **P < 0.01 and ***P < 0.001 versus the sham group; ^#^*P* < 0.05 and ^##^*P* < 0.01 versus the TAC group; NS: not significant. (**D**) Representative immunoblots showing ANP and BNP protein levels in the hearts of mice in the TAC group that were treated with vehicle, gallic acid, losartan, carvedilol, or furosemide. GAPDH was used as a loading control. Arrows indicate ANP and BNP bands, respectively. Representative images were cropped from different western blots. Larger images of the same blots are presented in Supplementary Figure Information. (**E**,**F**) ANP and BNP protein expression was quantified using densitometry. ***P < 0.001 versus the sham group; ^##^*P* < 0.01 versus the TAC group; NS: not significant.

To determine whether gallic acid suppresses the markers for heart failure, we performed real-time reverse transcription-polymerase chain reaction (RT-PCR) and western blotting. ANP, BNP, and skeletal α-actin are markers of cardiac hypertrophy and heart failure^19,20^. TAC significantly increased ANP, BNP, and skeletal α-actin (Fig. 2A‒C) mRNA levels in the heart tissue. Moreover, protein expressions of ANP and BNP were significantly increased in the heart tissues of mice in the TAC group than in the heart tissues of mice in the sham group (Fig. 2D–F). Treatment with gallic acid significantly decreased the expression of ANP and BNP mRNA and protein.

Beta myosin heavy chain (β-MHC) is another marker for heart failure. Alpha myosin heavy chain (α-MHC) changes to β-MHC in response to cardiac pathological conditions^21^. We observed that β-MHC mRNA levels were significantly increased in TAC hearts compared to sham hearts (Supplementary Fig. 3A). Treatment with gallic acid reduced the levels of β-MHC mRNA induced in the TAC group. In contrast, α-MHC expression was downregulated in TAC mice compared with the sham group, and was restored by gallic acid treatment (Supplementary Fig. 3B). Other drugs failed to regulate the shift of MHC. Thus, gallic acid treatment regulates expression of heart failure marker genes.

### Gallic acid reduces cardiac fibrosis in TAC-induced heart failure

**Figure 3:**
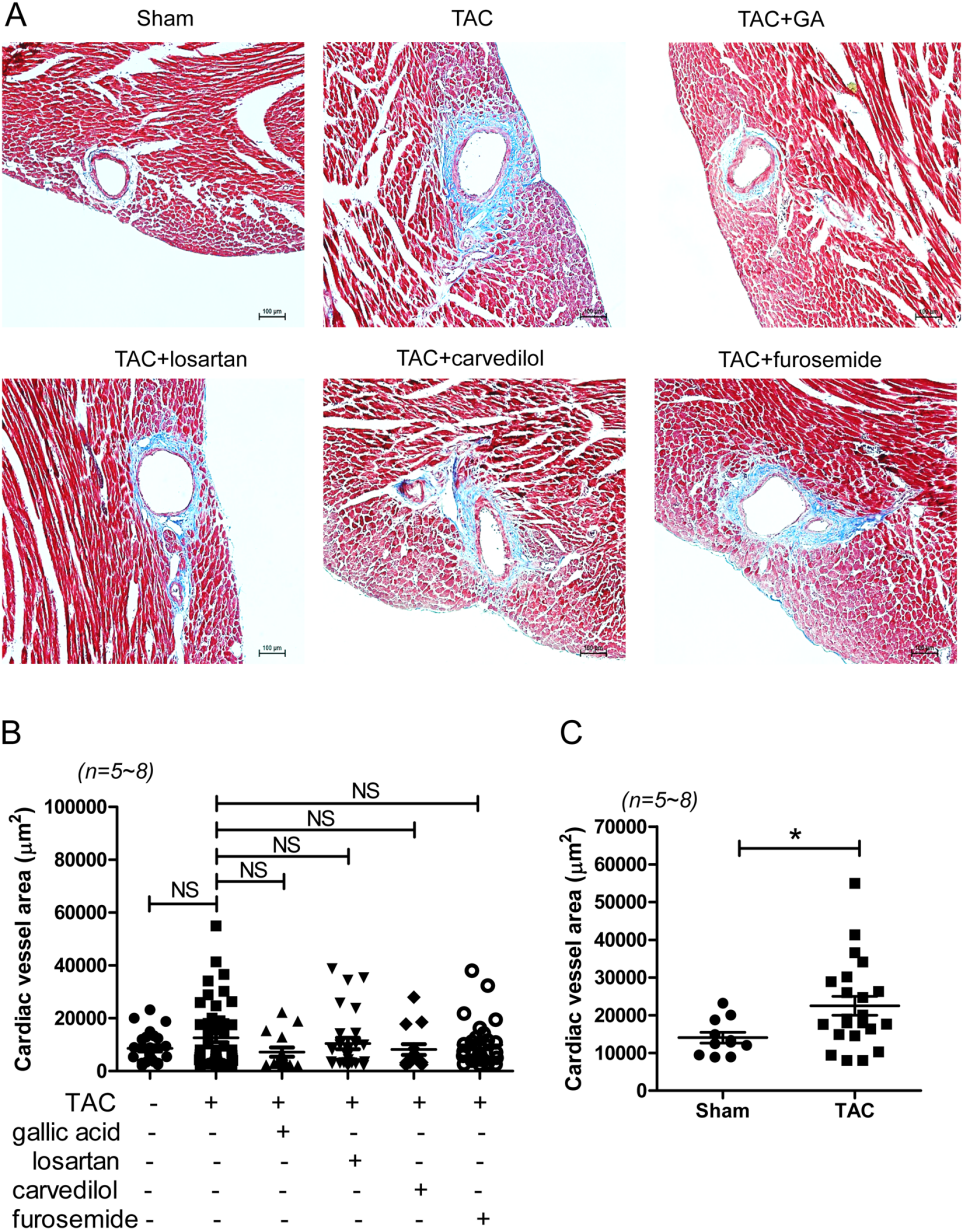
Gallic acid reduces perivascular fibrosis and vessel hypertrophy in TAC-induced heart failure. (**A**) Representative Trichrome II Blue staining of heart tissue sections of mice from the sham, TAC, TAC + gallic acid, TAC + losartan, TAC + carvedilol, and TAC + furosemide groups. Blue staining indicates collagen deposition. Scale bar = 100 µm (**B**) Measurements of cardiac vessel area in the heart tissues of mice in the TAC group treated with vehicle, gallic acid, losartan, carvedilol, or furosemide. NS indicates not significant. (**C**) Measurements of cardiac vessel area (more than 8,000 μm^2^) in the heart tissues from sham and TAC mice. *P < 0.05 versus the sham group.

Trichrome II Blue staining indicated increased perivascular fibrosis and vessel hypertrophy in the heart tissues of mice in the TAC group (Fig. 3A‒C). However, there was no definite interstitial fibrosis in the TAC group compared to the sham group. Gallic acid treatment reduced the perivascular fibrosis, but losartan, carvedilol, or furosemide did not affect it (Fig. 3A). No significant change was observed in vessel size when TAC mice were compared with mice treated with gallic acid, losartan, carvedilol, or furosemide (Fig. 3B). However, when we measured vessels >8,000 μm^2^, epicardial vessels were statistically larger in the hearts of mice in the TAC group than in those in the sham group (Fig. 3C).

**Figure 4:**
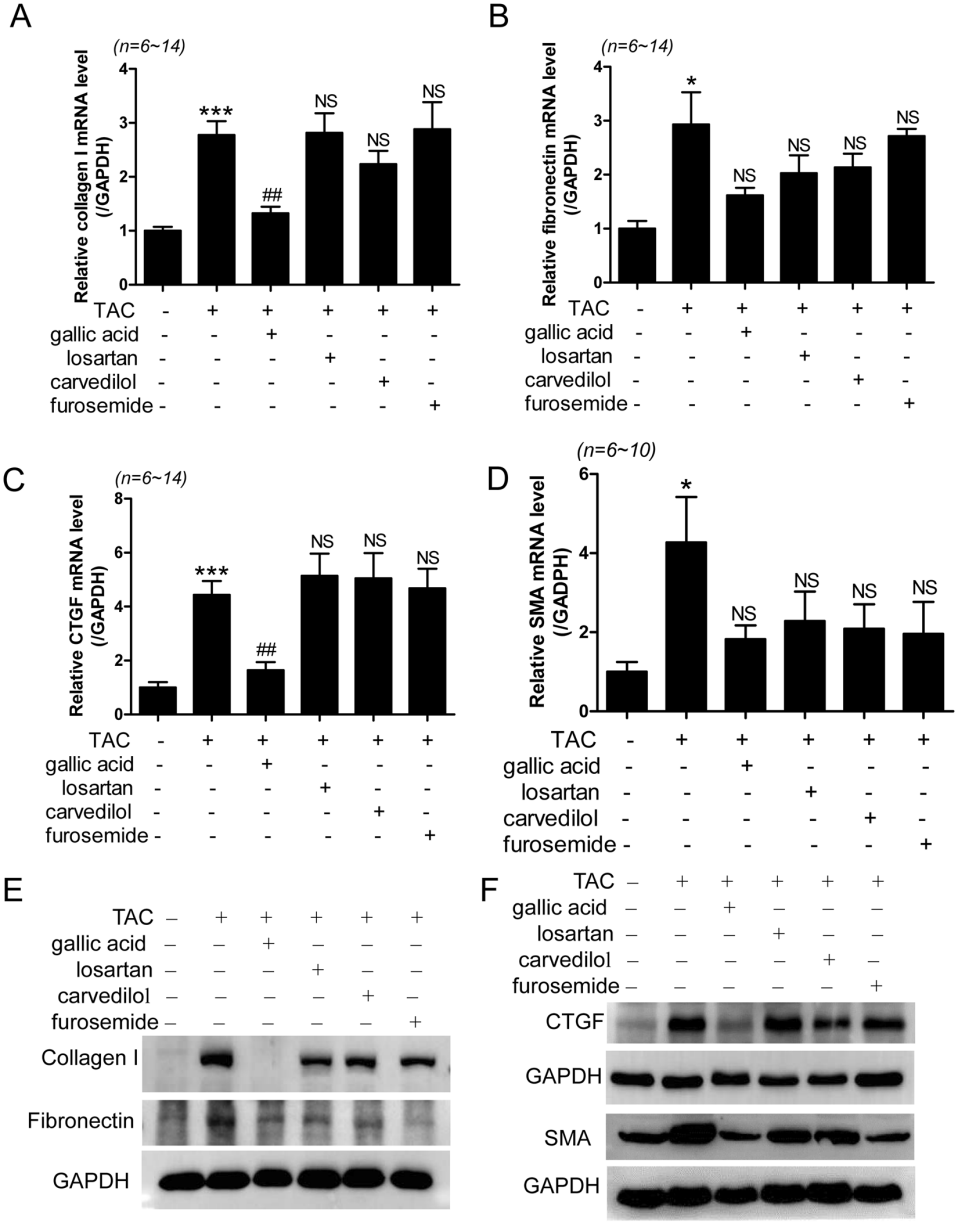
Gallic acid reduces expression of cardiac fibrosis marker-related genes in TAC-induced heart failure. Heart failure was induced 8 weeks after TAC surgery in mice, after which they were administered either gallic acid (100 mg/kg/day), losartan (3 mg/kg/day), carvedilol (1 mg/kg/day), or furosemide (3 mg/kg/day) for 2 weeks. (**A**–**D**) The mRNA levels of collagen type I, fibronectin, CTGF, and SMA were determined by RT-PCR. *P < 0.05, **P < 0.01, and ***P < 0.001 versus the sham group; ^##^*P* < 0.01 versus the TAC group; NS: not significant. (**E**,**F**) Representative immunoblots for collagen type I, fibronectin, CTGF, and SMA protein. GAPDH was used as a loading control. Representative western blot images were cropped from different parts of the same blots. Larger images of the same blots are presented in Supplementary Figure Information.

To further investigate whether gallic acid can reduce fibrosis markers in TAC-induced heart failure, we performed RT-PCR. The mRNA levels of collagen type I, fibronectin, CTGF, and SMA were significantly increased in the heart tissues of mice in the TAC group, compared to those in the heart tissues of mice in the sham group (Fig. 4A‒D). Treatment with gallic acid significantly reduced the expressions of collagen type I and CTGF mRNA (Fig. 4A and C). Treatment with gallic acid in TAC showed decreased expression tendency of fibronectin and SMA mRNA (Fig. 4B and D). Losartan, carvedilol, and furosemide treatment did not affect the expression of fibrosis marker gene (Fig. 4A‒D). Matrix metalloproteinase (MMP) affects the turnover of ECM proteins such as collagen type I. Helix-loop-helix protein p8 is required for induction of cardiac fibroblast MMP^22^. We determined the expression of MMPs in the hearts of mice in the TAC group that were treated with different drugs. The level of MMP2 mRNA was significantly increased in the hearts of mice in the TAC group compared to that in the hearts of mice in the sham group. The level was significantly reduced by treatment with gallic acid (Supplementary Fig. 4A), while the other drugs did not affect it. Gallic acid and other drugs did not reduce the levels of MMP9 mRNA in TAC mice (Supplementary Fig. 4B). In addition, they did not affect the MMP13 mRNA levels in TAC mice (Supplementary Fig. 4C).

The level of p8 was also significantly increased in the hearts of mice in the TAC group compared to that in the hearts of mice in the sham group, and this induction was significantly reduced by administration of gallic acid (Supplementary Fig. 4D). We determined the protein expression of fibrosis-related genes. Western blotting showed that treatment with gallic acid reduced the expression of collagen type I and CTGF protein in the heart tissues of rats in the TAC group (Fig. 4E,F and Supplementary Fig. 5A and C). The fibronectin protein level was significantly increased in the TAC hearts compared to the sham hearts (Fig. 4E and Supplementary Fig. 5B). However, all drugs, including gallic acid, did not statistically reduce fibronectin protein levels (Supplementary Fig. 5B). Similarly, SMA protein levels were increased in TAC hearts compared to sham hearts; however, they were not significantly reduced by drugs (Fig. 4F and Supplementary Fig. 5D). To determine the regulatory mechanism of gallic acid in fibrosis, we investigated the Smad signaling pathway. Smad3 protein was phosphorylated in response to TAC stimuli. Gallic acid treatment reduced phosphorylated Smad3 expression. Moreover, the protein expression of unphosphorylated Smad3 was significantly increased in TAC hearts compared to sham hearts, and was reduced by gallic acid treatment, but not by other drugs (Supplementary Fig. 6A and B).

### Gallic acid suppresses TGF-β1-induced fibrosis in rat cardiac fibroblast cells

**Figure 5:**
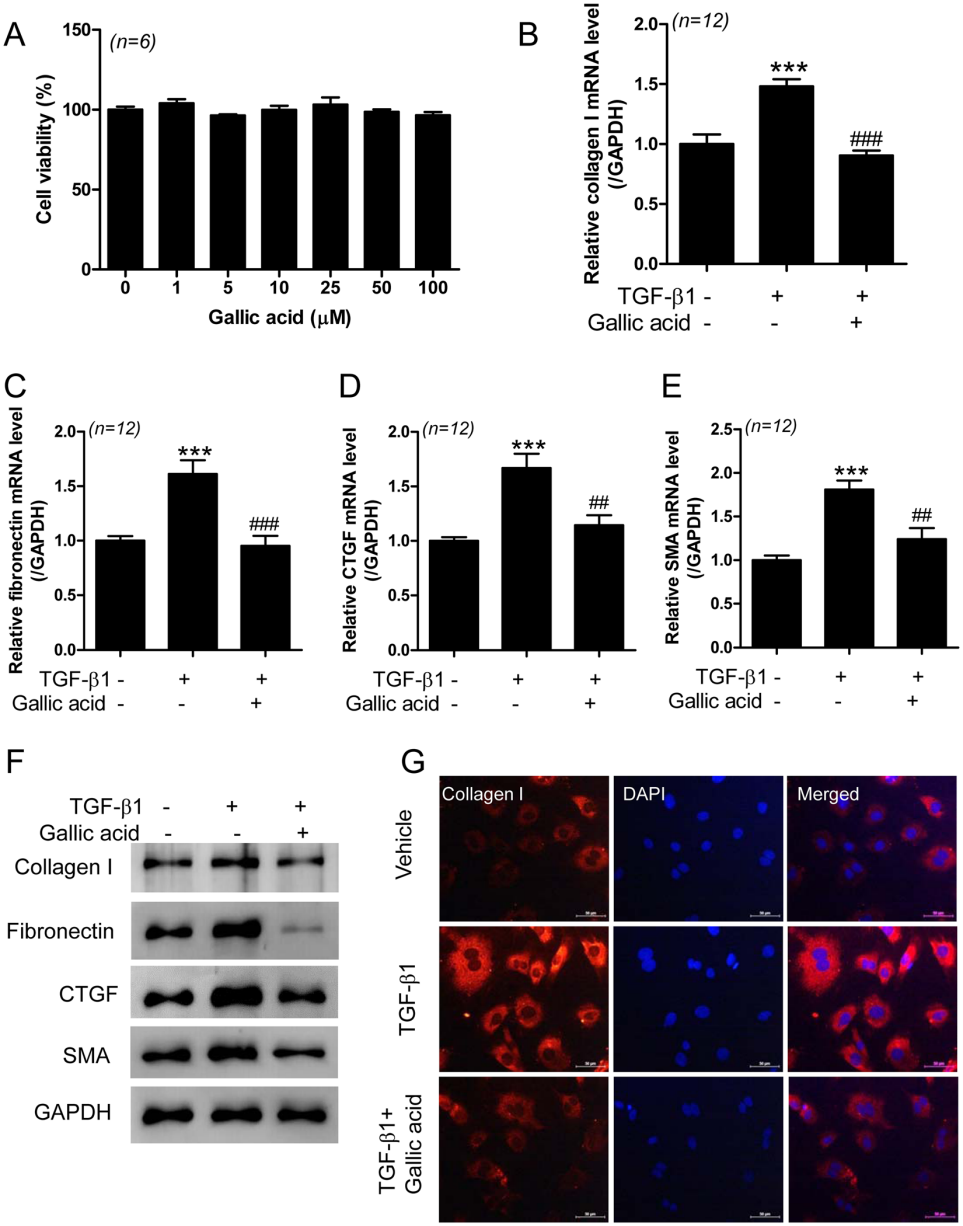
Gallic acid suppresses TGF-β1-induced fibrosis marker genes in rat cardiac fibroblast cells. (**A**) Rat neonatal cardiac fibroblast cells were treated with the indicated concentrations of gallic acid for 24 h, and cell viability was assessed by MTT assay. (**B**–**G**) Rat neonatal cardiac fibroblast cells were serum starved (0.5% FBS DMEM) overnight, after which TGF-β1 (5 ng/mL) was added for 3 h, and then the cells were treated with gallic acid for 9 h. The cells were used at passage 1‒2. The transcript levels for collagen type I (**B**), fibronectin (**C**), CTGF (**D**), and SMA (**E**) were normalized to GAPDH mRNA using RT-PCR. (**F**) Representative immunoblots for collagen I, fibronectin, CTGF, and SMA. Representative western blot images were cropped from different parts of the same blots. Larger images of the same blots are presented in Supplementary Figure Information. (**G**) Representative immunofluorescence images for collagen type I in rat cardiac fibroblasts. ICC staining was performed using anti-collagen type I antibody (red). DAPI was used to stain cell nuclei (blue). Scale bar = 50 µm.

TGF-β1 is a major regulator in the process of fibrosis. We investigated the expression of fibrosis marker gene induced by TGF-β1 in rat neonatal cardiac fibroblast cells. The mRNA levels of collagen type I, fibronectin, CTGF, and SMA were significantly increased in response to TGF-β1 stimulus in a dose-dependent manner (Supplementary Fig. 7A‒D). To determine the cell cytotoxicity of gallic acid, we performed 3-(4,5-dimethylthiazol-2-yl)-2,5-diphenyltetrazolium bromide (MTT) assay. Gallic acid showed no cytotoxicity up to a concentration of 100 µM (Fig. 5A). Treatment with gallic acid significantly reduced TGF-β1-induced collagen type I, fibronectin, CTGF, and SMA transcript levels in rat cardiac fibroblast cells (Fig. 5B‒E). We observed that gallic acid inhibits the protein expressions of collagen I, fibronectin, CTGF, and SMA induced by TGF-β1 in fibroblast cells (Fig. 5F and Supplementary Fig. 8A‒D). To further prove the antifibrotic effect of gallic acid, we performed immunocytochemistry (ICC) in rat neonatal cardiac fibroblasts. Fluorescence ICC revealed the enhanced expression of collagen type I after TGF-β1 treatment in cardiac fibroblasts. The increased level was dramatically decreased by treatment with gallic acid (Fig. 5G). We confirmed these results by measuring the fluorescence intensity of collagen type I in the presence or absence of gallic acid with TGF-β1 stimulation (Supplementary Fig. 9).

## Discussion

The present study demonstrates that gallic acid improves cardiac dysfunction and fibrosis in a mouse model of pressure overload-induced heart failure and in primary rat cardiac fibroblasts. In this study, heart failure and fibrosis manifested within 10 weeks of TAC in mice. The lumen of the hearts of mice in the TAC group was significantly enlarged, leading to a decrease in FS, indicating cardiac dysfunction. Heart failure is highly fatal owing to the associated high mortality, rendering its treatment important. Clinical drugs, including ARBs, beta-blockers, and diuretics did not improve cardiac dysfunction and fibrosis in mice in the TAC group. Interestingly, gallic acid improved cardiac dysfunction, as determined by echocardiography. We were unable to explain the exact mechanism by which gallic acid reduced the increased left ventricular diameter in the hearts of mice in the TAC group. Left ventricular diameter is much more important than left ventricular mass with regard to cardiac mortality, and it is useful to assess the risk of sudden cardiac death^23^. In addition, the Studies of Left Ventricular Dysfunction (SOLVD) registry showed that an increase in LVESD is associated with cardiovascular death^24^. Our valuable finding is, to the best of our knowledge, the first evidence that administration of gallic acid ameliorates heart failure and cardiac fibrosis. Similarly, Ramezani-Aliakbari *et al*. reported that gallic acid improved left ventricular dysfunction in a rat model of alloxan-induced diabetes mellitus^25^. Advanced glycation end products (AGEs) are implicated in cardiovascular diseases in diabetics. AGE-induced cardiac fibrosis and remodeling was prevented by gallic acid administration^26^. Furthermore, pretreatment with gallic acid improved doxorubicin-induced electrocardiographic abnormalities and cardiac damage^27^. Antioxidant effects of gallic acid mitigated diazinone-induced cardiovascular dysfunction in a rat animal model^28^ and cyclophosphamide-induced cardiorenal dysfunction^29^. Gallic acid was reported to have cardioprotective effects following exposure to various substances such as aluminum oxide^30^, lindane^31^, and isoproterenol^32^.

Recently, we reported that gallic acid attenuates pulmonary fibrosis in a mouse model of TAC-induced heart failure^17^. In the present study, gallic acid significantly reduced the levels of heart failure markers including ANP, BNP, skeletal α-actin, and β-MHC in the hearts of mice in the TAC group. In contrast, losartan, carvedilol, or furosemide did not decrease the mRNA and protein expression of ANP, BNP, and skeletal α-actin. In our previous study, we observed that gallic acid prevents isoproterenol-induced cardiac hypertrophy and fibrosis^14^. In addition, gallic acid reduced left ventricular hypertrophy in rats with spontaneous hypertension^16^, and it diminished left ventricular remodeling in N^G^-nitro-L-arginine methyl ester (L-NAME)-induced hypertension^15^. These observations imply that gallic acid regulates cardiac remodeling. In our experimental conditions, losartan and carvedilol were not effective for cardiac dysfunction, which may be attributed to the low doses of losartan (3 mg/kg/day) and carvedilol (1 mg/kg/day). For example, Wang *et al*. reported that losartan (13.4 mg/kg/day) attenuated the development of cardiac hypertrophy and heart failure in TAC for 4 weeks^33^. This study used higher-dose losartan than that used in our study. In addition, losartan was considered to have a therapeutic effect on the heart because the drug administration period of this study was twice as long as that of ours. Hampton *et al*. reported that a high dose of carvedilol (30 mg/kg/day) improved cardiac performance^34^.

Cardiac fibrosis and excessive accumulation of ECM proteins are the major hallmarks of pathological cardiac remodeling in heart failure, and cardiac fibrosis therapy may be involved in the recovery of cardiac dysfunction. In the present study, treatment with gallic acid reduced cardiac fibrosis in a mouse model of pressure overload-induced heart failure. Gallic acid reduced the deposition of collagen in the perivascular regions in the heart tissues of mice in the TAC group. Our TAC model had no clear interstitial fibrosis. Mice with chronic heart failure induced by 10 weeks of TAC had larger vessels than those of sham mice, as determined by Trichrome II Blue staining and cardiac vessel area. The presence of many large vessels (>8,000 μm^2^) in the hearts of mice in the TAC group may be attributed to the supply of nutrition and oxygen through blood to the heart muscle. These hearts also had many small vessels.

Treatment with gallic acid significantly reduced the mRNA and protein expressions of collagen type I and CTGF in the hearts of mice in the TAC group. In contrast, administration of losartan, carvedilol, or furosemide did not have any beneficial effect on the development of fibrosis, which may be attributable to the use of low-dose losartan, carvedilol, and furosemide in the TAC model. Among MMPs, gallic acid decreased MMP2 mRNA level in the hearts of mice in the TAC group. In addition, treatment with gallic acid significantly reduced p8 mRNA levels in response to TAC. Cardiac fibroblasts play an important role in the process of fibrosis, and they are responsible for homeostasis of the ECM^5^. Cardiac fibroblasts get converted to activated cardiac myofibroblasts in response to pathological stress. SMA is a marker of activated myofibroblasts. In our study, SMA mRNA and protein levels were significantly increased *in vivo* and *in vitro*. We clearly demonstrated that treatment with gallic acid attenuates fibrosis marker genes in TGF-β1-treated rat cardiac fibroblasts. ICC showed a definitely reduced expression of collagen type I. Our previous report demonstrated that gallic acid suppresses cardiac fibrosis in a hypertension model of L-NAME through downregulation of histone deacetylase 2^15^. In addition, gallic acid reduced cardiac fibrosis in response to isoproterenol through downregulation of phospho-Smad3 and its binding activity to collagen promoter^14^. In the current study, the regulatory mechanism by which gallic acid reduces fibrosis may be attributed to the reduction of Smad3 protein levels induced by TAC. This result is consistent with a previous report that the protein level of Smad3 was increased at week 8 after TAC^35^.

Thus far, gallic acid has been reported to have pleiotropic beneficial effects on diabetes^36,37^, cancer^38–40^, cardiac hypertrophy and fibrosis^14^, pulmonary fibrosis^17^, vascular calcification^13^, and hypertension^16^. In the present study, we added a novel therapeutic effect of gallic acid for heart failure.

In summary, we demonstrated that administration of gallic acid improves cardiac dysfunction and fibrosis in a mouse model of pressure overload-induced heart failure. Gallic acid showed better efficacy against cardiac dysfunction and fibrosis than losartan, carvedilol, and furosemide did, and reduced the risk of heart failure and fibrosis *in vivo* and *in vitro*. Thus, we suggest that gallic acid could be a novel therapeutic agent for treatment of heart failure with fibrosis.

## Electronic supplementary material

Supplementary Figures
