# Supplementary material for: Gallic acid improves cardiac dysfunction and fibrosis in pressure overload-induced heart failure
Source: Sci Rep. 2018 Jun 18;8:9302. doi: 10.1038/s41598-018-27599-4 (PMC6006337; doi:10.1038/s41598-018-27599-4)

Gallic acid improves cardiac dysfunction and fibrosis in pressure overload-induced heart failure

Li Jin[1,2,+], Simei Sun[1,3,4+], Yuhee Ryu[1,4+], Zhe Hao Piao[5], Bin Liu[5], Sin Young Choi[1,3,4], Gwi Ran Kim[1,4], Hyung-Seok Kim[6], Hae Jin Kee[1,4*], and Myung Ho Jeong[1,4*]

[1]Heart Research Center of Chonnam National University Hospital, Gwangju 61469, Republic of Korea

[2]Changchun Central General Hospital, 1810 Renmin St, Nanguan Qu, Changchun Shi, Jilin Sheng, 130051, China

[3]Molecular Medicine, BK21 plus, Chonnam National University Graduate School, Gwangju 61469, Republic of Korea

[4]Hypertension Heart Failure Research Center, Chonnam National University Hospital, Gwangju 61469, Republic of Korea

[5]The Second Hospital of Jilin University, Changchun, Jilin 130041, China

[6]Department of Forensic Medicine, Chonnam National University Medical School, Gwangju 61469, Republic of Korea

Supplementary Figures

Supplementary Fig. 1. Mice exhibited phenotype of heart failure at 8 weeks after TAC surgery.

Mice were subjected to TAC or sham operations. Echocardiography was performed at 2, 4, 6, and 8 weeks after the TAC operation to ensure heart failure. Cardiac dysfunction was observed at 8 weeks, with an increase in LVESD (**A)** and LVEDD (**B**) and decrease in FS (**C**). **$P < 0.01$ and ***$P < 0.001$ versus the sham group. Levels of significance were tested using Student's t-test.

Supplementary Fig. 2. Gallic acid restored heart rate in response to TAC.

Mice were subjected to TAC or sham operations. After 8 weeks, mice were administered drugs for 2 weeks. Heart rate was measured using echocardiography. ***$P < 0.001$ versus the sham group; $^{###}P < 0.001$ versus the TAC group; NS: not significant

Supplementary Fig. 3. Gallic acid regulates the shift from α-MHC to β-MHC mRNA levels in response to TAC.

After 8 weeks of TAC treatment, gallic acid, losartan, carvedilol, and furosemide were intraperitoneally administered daily to mice for 2 weeks. Transcription levels are shown for β-MHC and α-MHC in mice from the sham, TAC, and TAC treated with gallic acid, losartan, carvedilol, and furosemide groups. *$P < 0.05$ and ***$P < 0.001$ versus the sham group; $^{\#}P <$

0.05 and $^{##}P < 0.01$ versus the TAC group; NS: not significant

Supplementary Fig. 4. Gallic acid suppresses MMP2 and p8 mRNA levels in the hearts of mice in the TAC group

After 8 weeks of TAC treatment, gallic acid, losartan, carvedilol, and furosemide were intraperitoneally administered daily to mice for 2 weeks. (**A–D**) Transcript levels for MMP2, MMP9, MMP13, and p8 from mice from the sham, TAC, and TAC treated with gallic acid, losartan, carvedilol, and furosemide groups.

$*P < 0.05$ versus the sham group; $^{#}P < 0.05$ versus the TAC group; NS: not significant

Supplementary Fig. 5. Gallic acid reduces protein expression of collagen type I and CTGF induced by TAC.

After 8 weeks of TAC treatment, gallic acid, losartan, carvedilol, and furosemide were intraperitoneally administered daily to mice for 2 weeks. (**A-D**) Western blot images of collagen type I, fibronectin, CTGF, and SMA from different groups were quantified by densitometry. $*P < 0.05$ and $**P < 0.01$ versus the sham group; $^{#}P < 0.05$ versus the TAC group; NS: not significant

Supplementary Fig. 6. Gallic acid reduced the protein expression of Smad3 induced by TAC.

After 8 weeks of TAC treatment, gallic acid, losartan, carvedilol, and furosemide were intraperitoneally administered daily to mice for 2 weeks. (**A**) Western blot images of p-

Smad3 and Smad3 from different groups are shown. Smad3 protein was quantified by densitometry. ***$P < 0.001$ versus the sham group; $^{\#\#}P < 0.01$ versus the TAC group; NS: not significant

Supplementary Fig. 7. Fibrosis marker genes are increased in response to TGF-β1 stimuli in rat cardiac fibroblasts.

Rat cardiac fibroblasts were serum starved overnight and incubated with TGF-β1 at indicated time points. Transcript levels for collagen type I, fibronectin, CTGF, and SMA were determined using qRT-PCR.

*$P < 0.05$, **$P < 0.01$, and ***$P < 0.001$ versus vehicle-treated group

Supplementary Fig. 8. Gallic acid reduces protein expression of fibrosis marker genes in TGF-β1-treated cardiac fibroblasts

Rat cardiac fibroblasts were serum starved overnight and treated with vehicle or gallic acid in the presence of TGF-β1. Protein levels for collagen type I, fibronectin, CTGF, and SMA were determined using qRT-PCR.

Supplementary Fig. 9. Gallic acid reduced collagen type I expression in TGF-β1-treated cardiac fibroblasts.

Rat cardiac fibroblasts were serum starved overnight and treated with vehicle or gallic acid in the presence of TGF-β1. After image acquisition under the same exposure conditions, the

fluorescence intensity of collagen type I was quantified in the attached images. $^{***}P < 0.001$ versus the vehicle group; $^{###}P < 0.001$ versus the TGF-β1 group.

Supplementary Figure 1

A

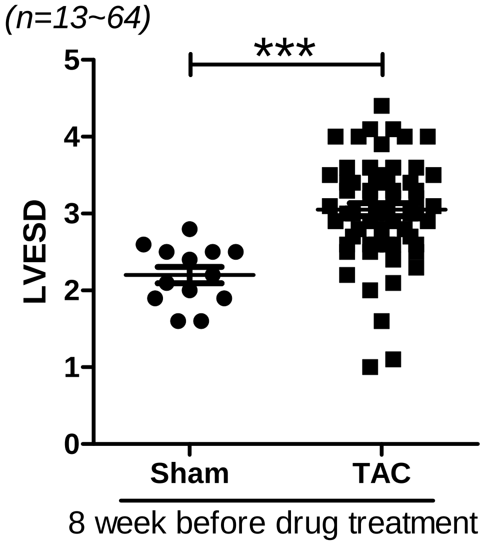

B

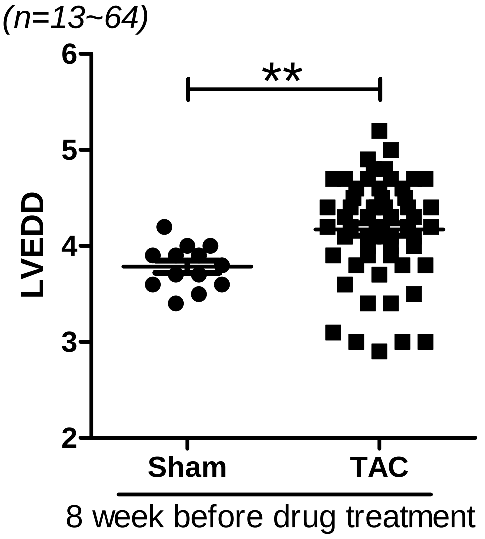

C

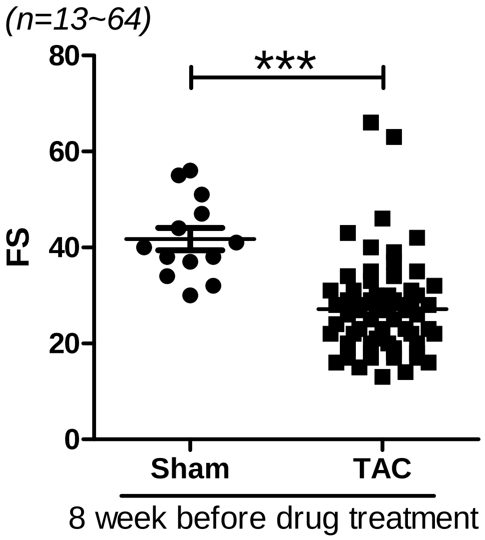

Supplementary Figure 2

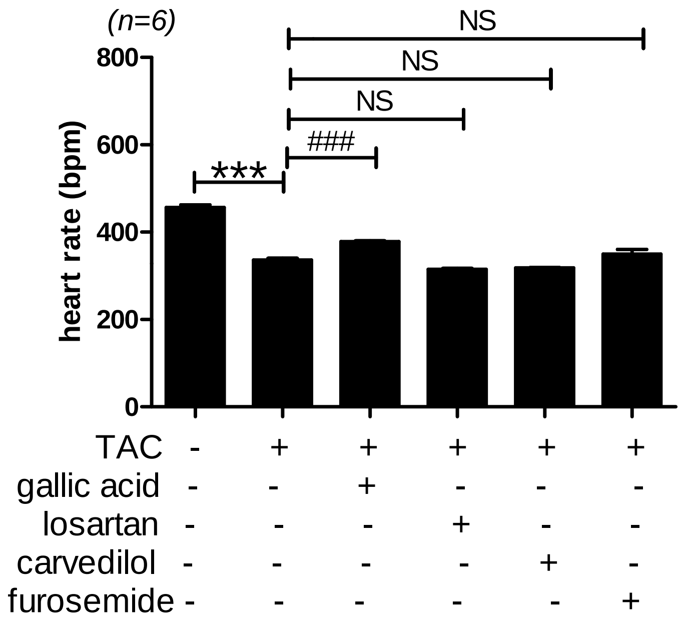

Supplementary Figure 3

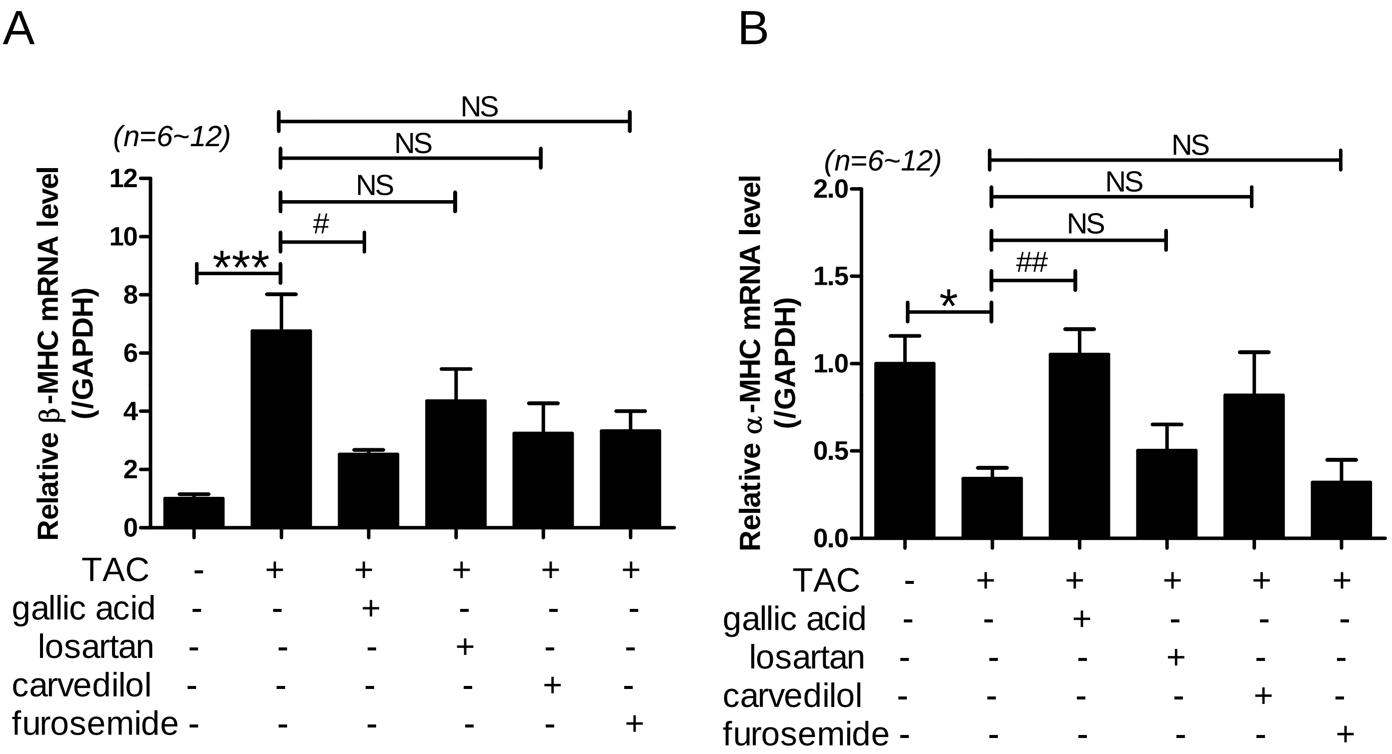

Supplementary Figure 4

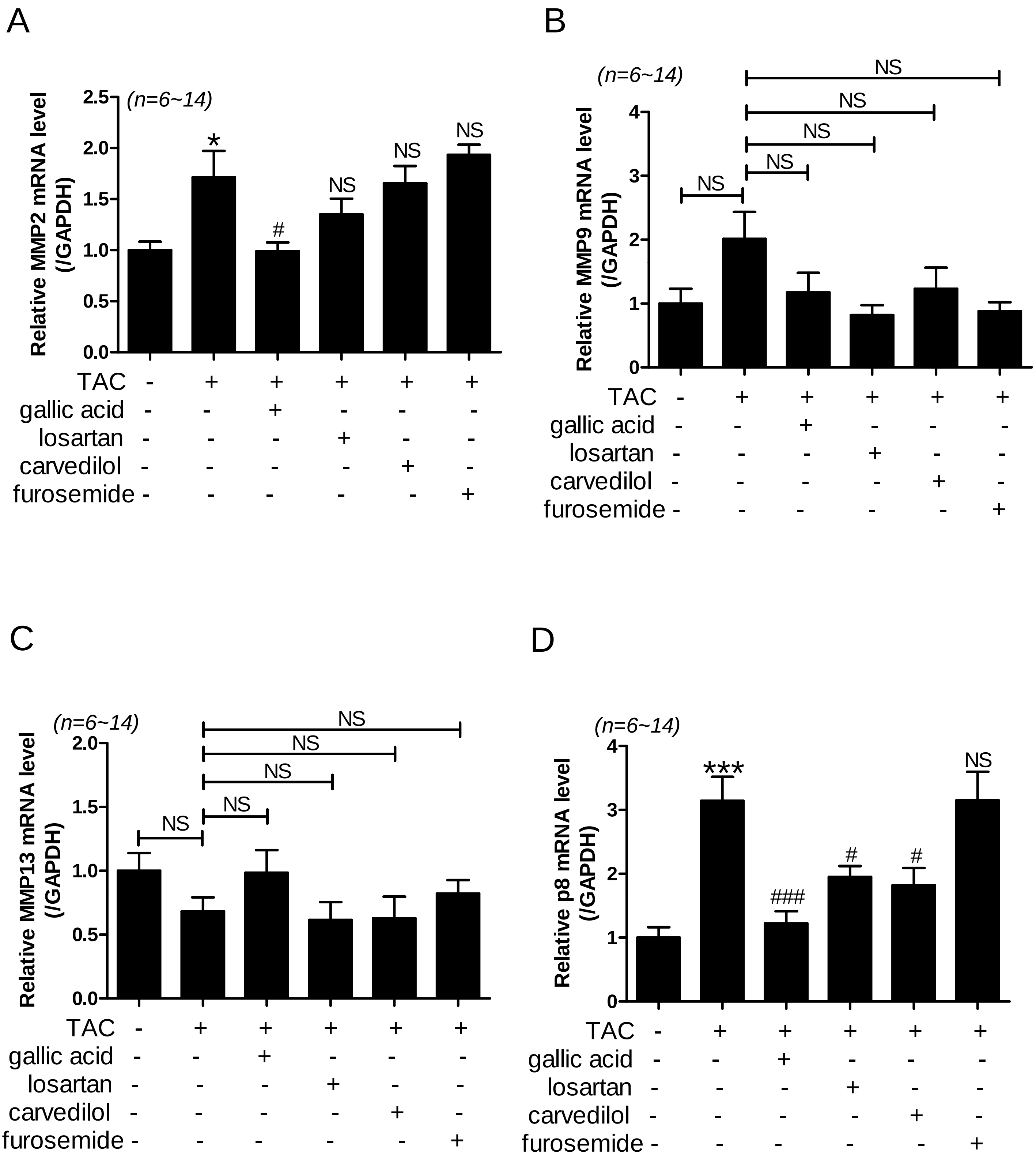

Supplementary Figure 5

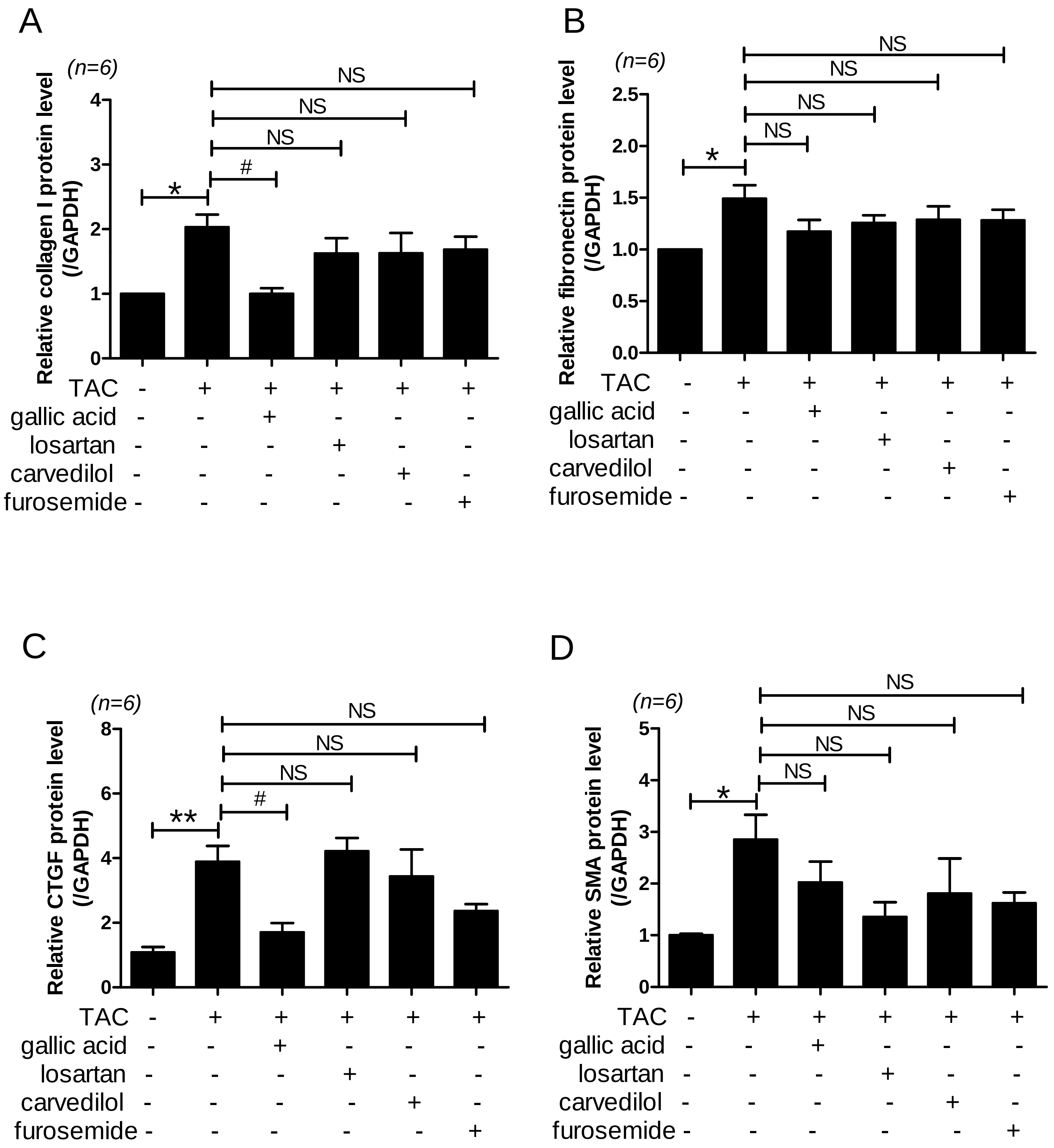

Supplementary Figure 6

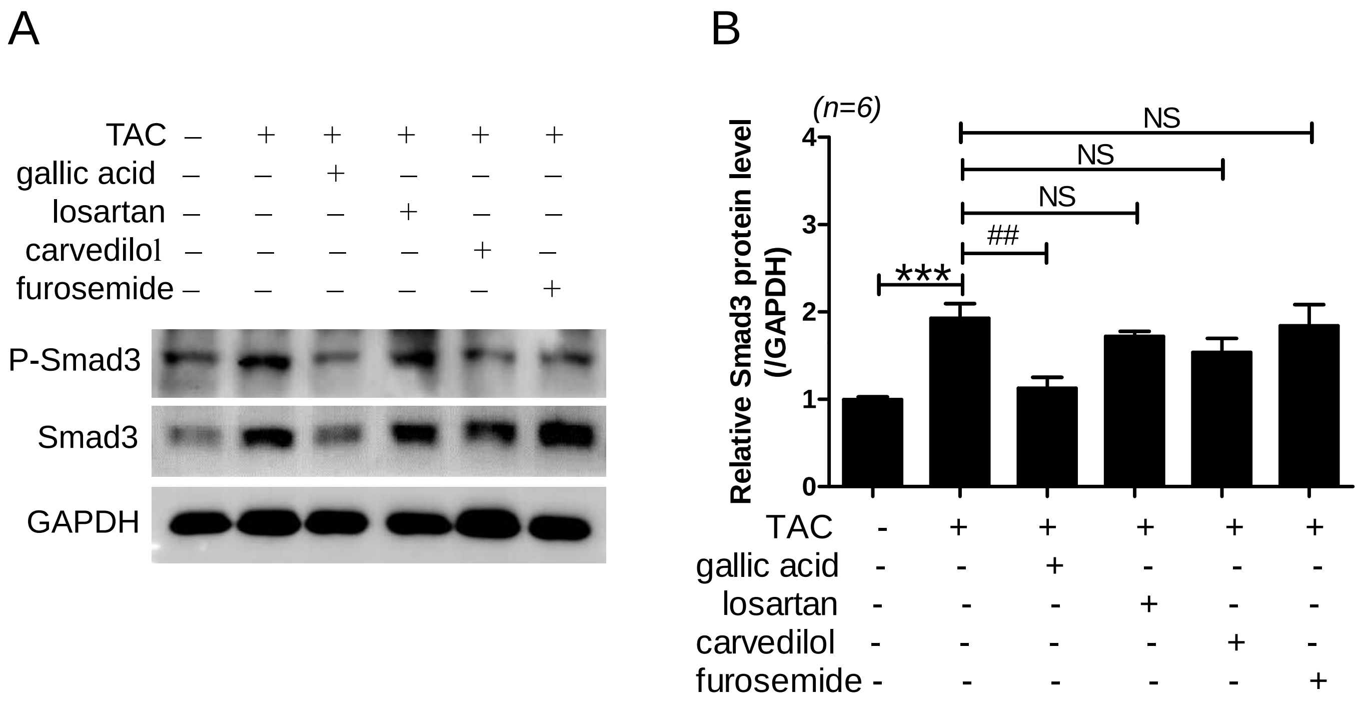

Supplementary Figure 7

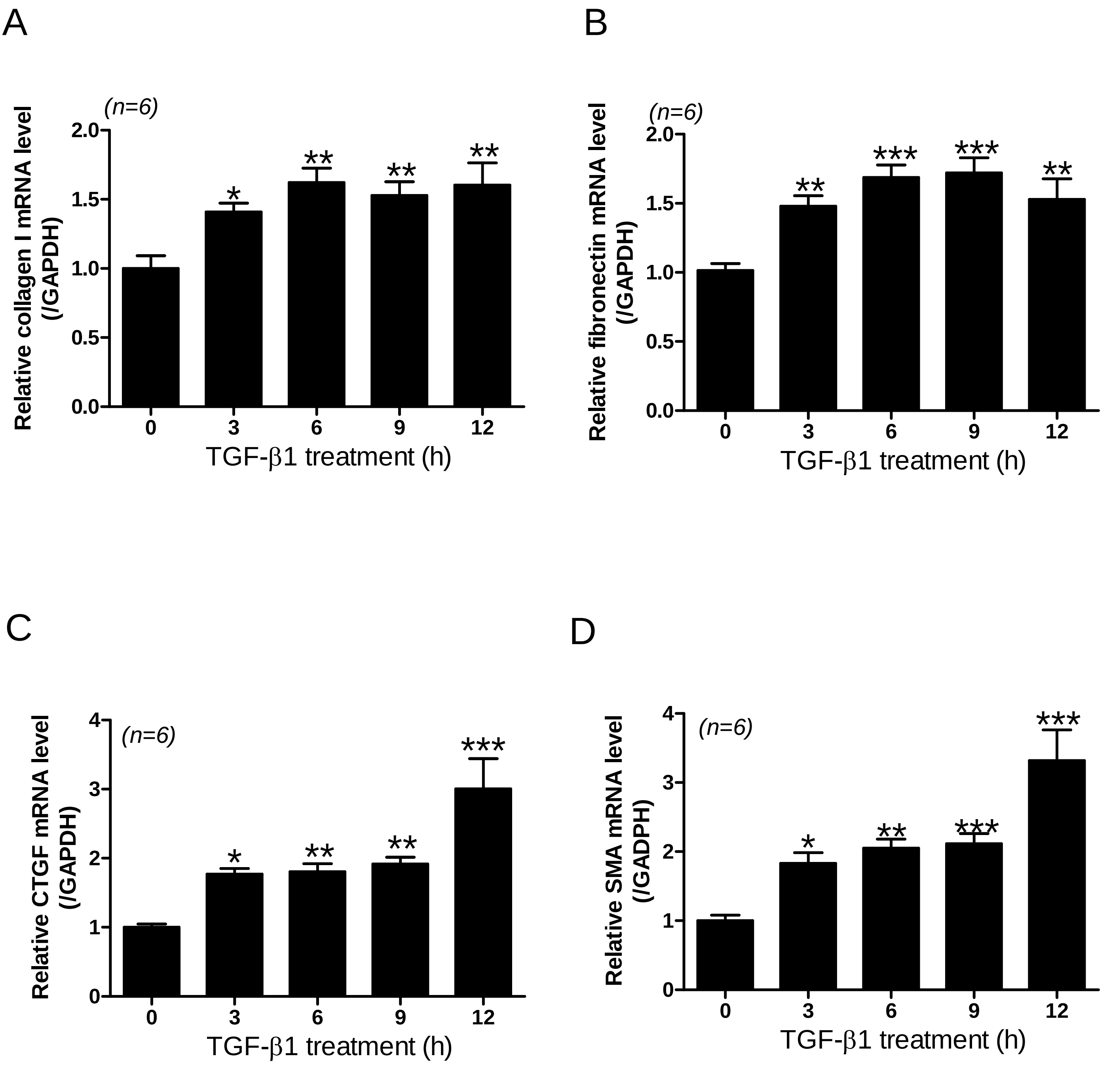

Supplementary Figure 8

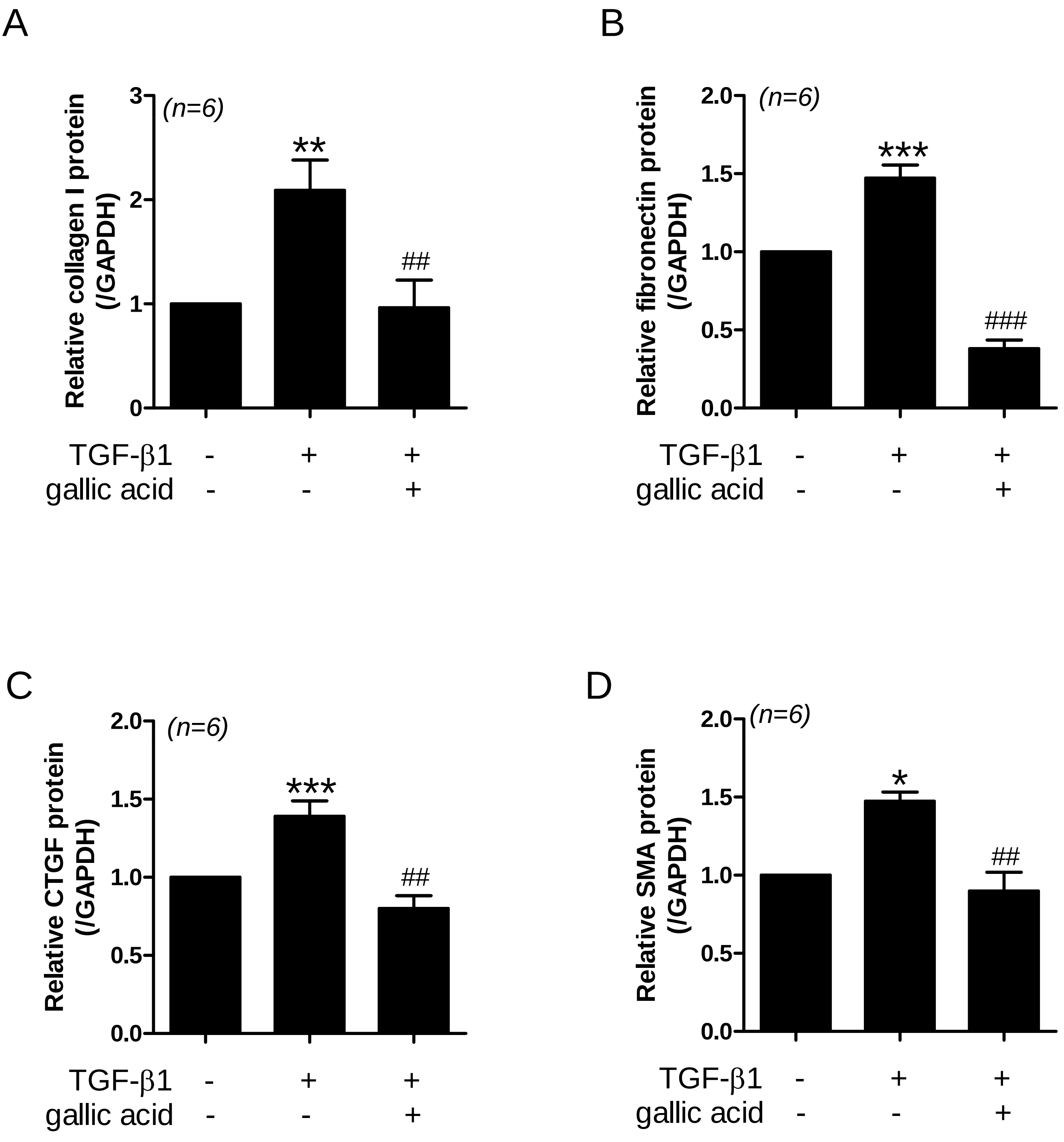

Supplementary Figure 9

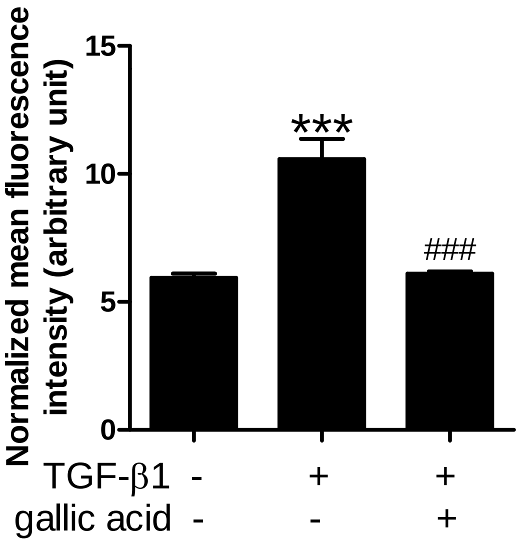

Supplementary information (Figure 2D)

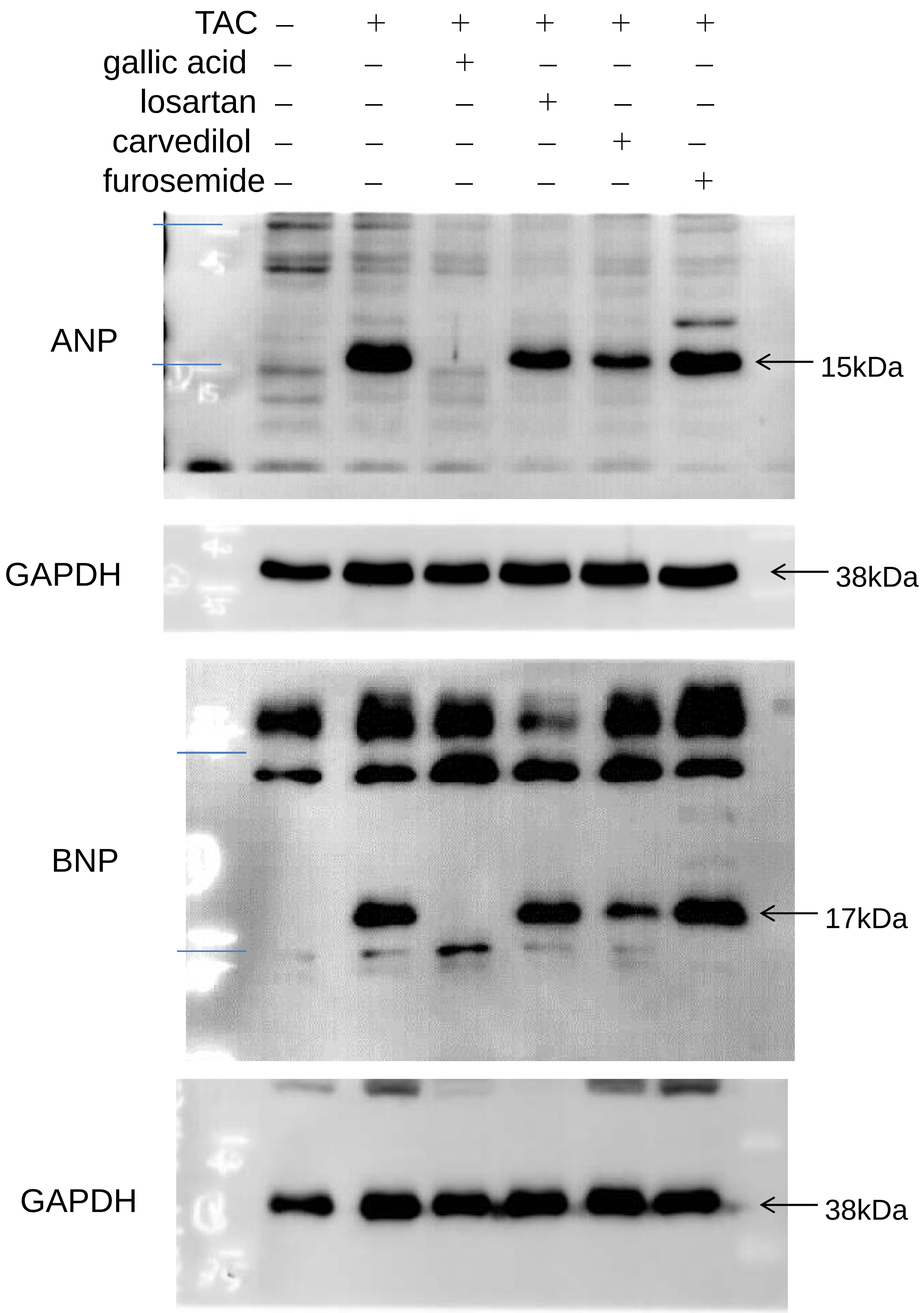

Supplementary information (Figure 4E)

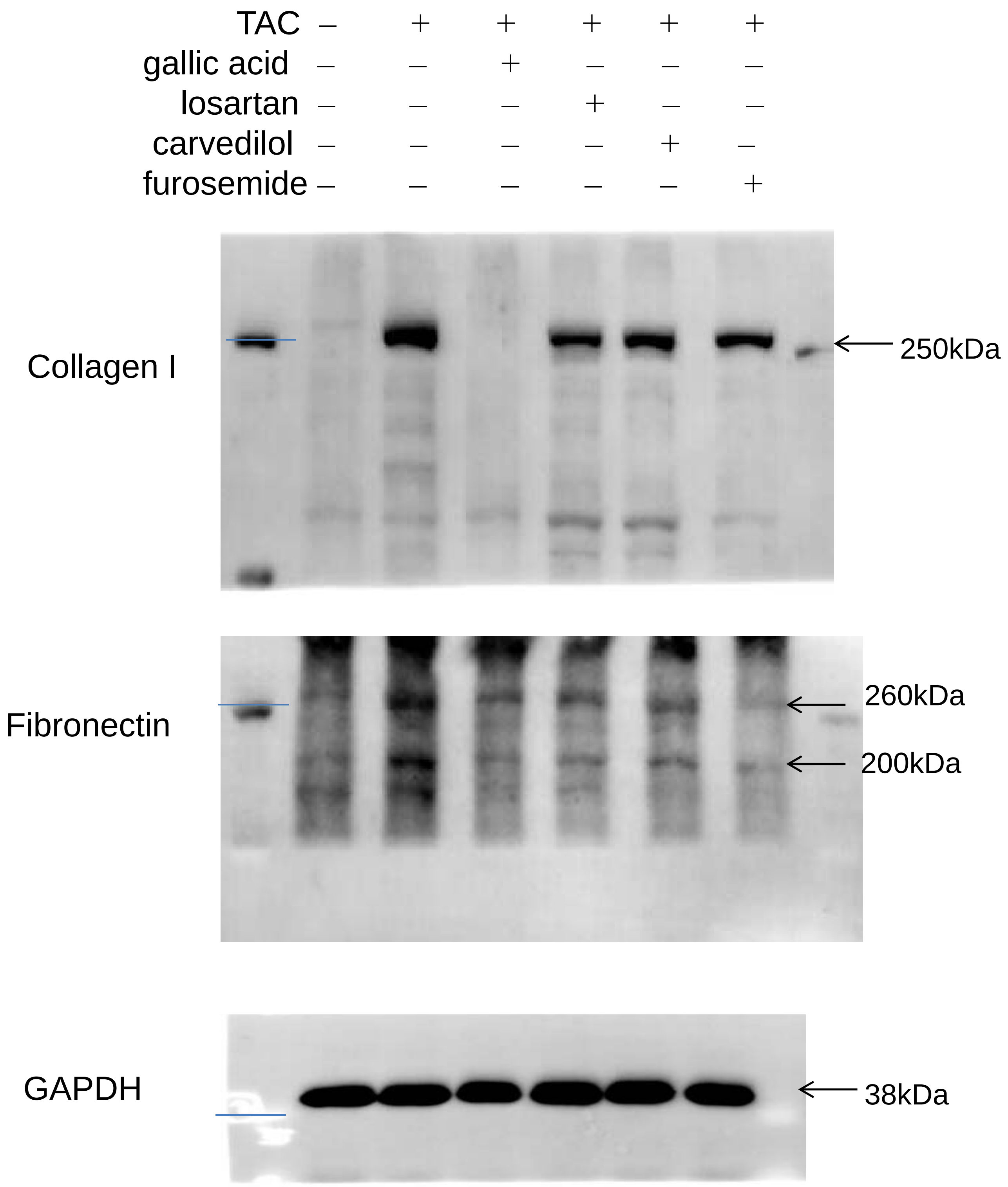

Supplementary information (Figure 4F)

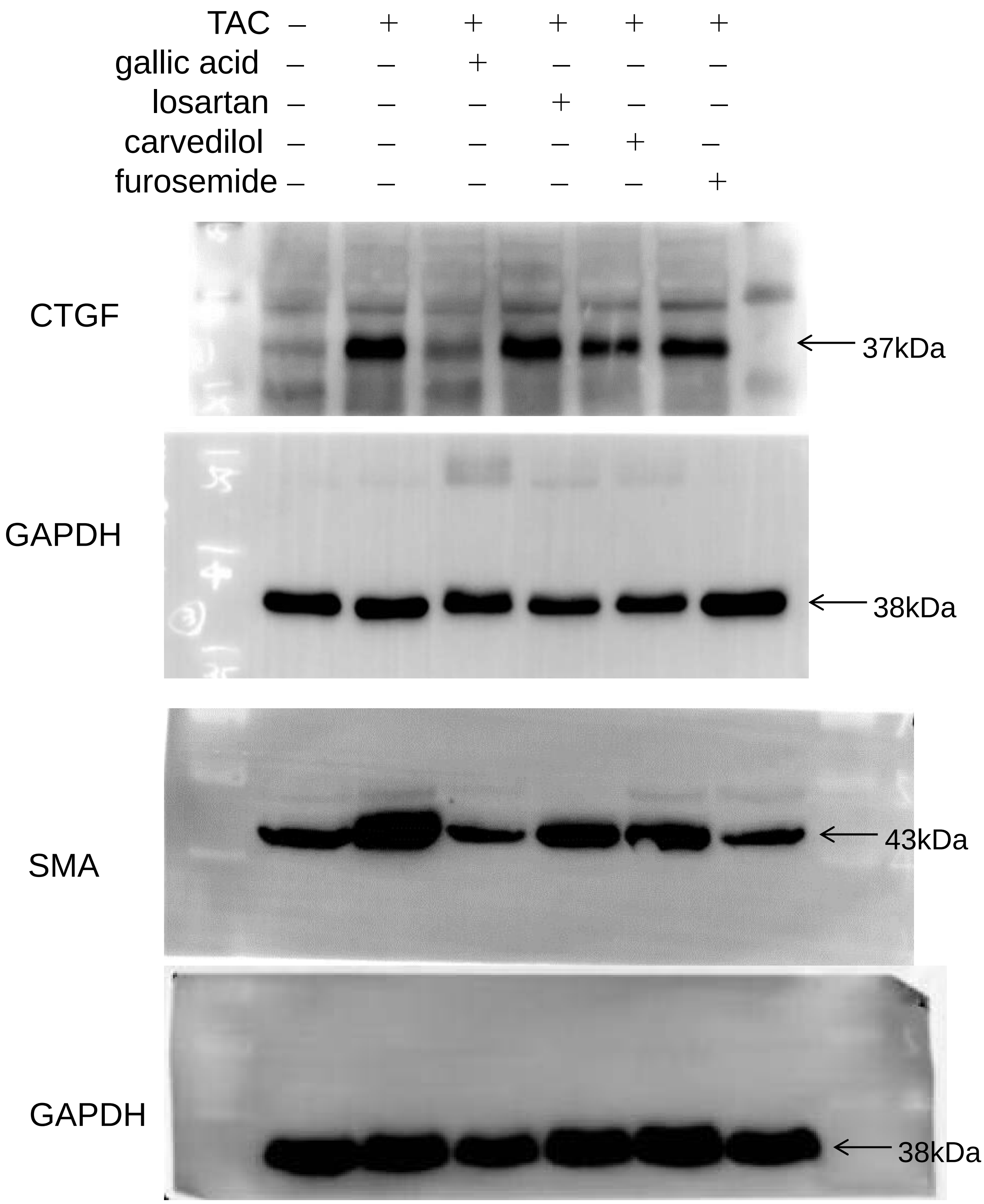

Supplementary information (Figure 5F)

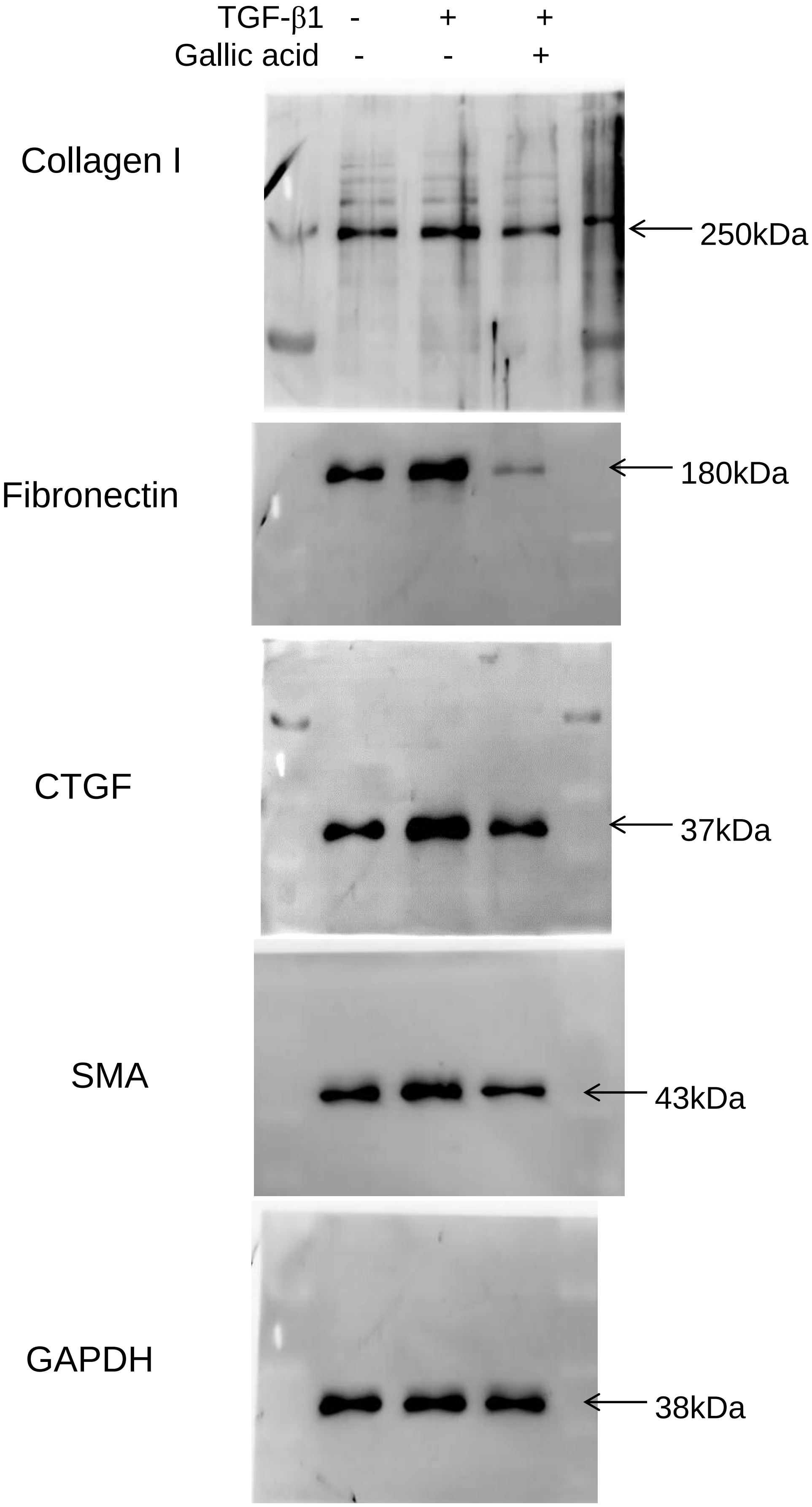

Supplement: Supplementary file 1 — Supplementary Figures [file 41598_2018_27599_MOESM1_ESM.pdf]
